# Evaluation of the Transformation and Leaching Behavior of Two Polyfluoroalkyl Phosphate Diesters in a Field Lysimeter Study

**DOI:** 10.1021/acs.jafc.2c03334

**Published:** 2022-11-02

**Authors:** René Lämmer, Eva Weidemann, Bernd Göckener, Thorsten Stahl, Jörn Breuer, Janine Kowalczyk, Hildegard Just, Runa S. Boeddinghaus, Matthias Gassmann, Hans-Willi Kling, Mark Bücking

**Affiliations:** † Department of Environmental and Food Analysis, Fraunhofer Institute for Molecular Biology and Applied Ecology IME, Auf dem Aberg 1, 57392 Schmallenberg, Germany; ‡ Department of Hydrology and Substance Balance, University of Kassel, Kurt-Wolters-Straße 3, 34125 Kassel, Germany; § Chemical and Veterinary Analytical Institute Münsterland-Emscher-Lippe, Joseph-König-Straße 40, 48147 Münster, Germany; ∥ Center for Agricultural Technology Augustenberg (LTZ), Neßlerstraße 25, 76227 Karlsruhe, Germany; ⊥ German Federal Institute for Risk Assessment, Max-Dohrn-Straße 8-10, 10589 Berlin, Germany; # Department of Chemistry and Biology, University of Wuppertal, Gaußstraße 20, 42119 Wuppertal, Germany; ¶ School of Clinical Sciences at Monash Health, Faculty of Medicine, Nursing and Health Sciences, Monash University, Clayton 3800, Victoria, Australia

**Keywords:** PFAS, diPAPs, lysimeter, transformation, precursor, PFCAs

## Abstract

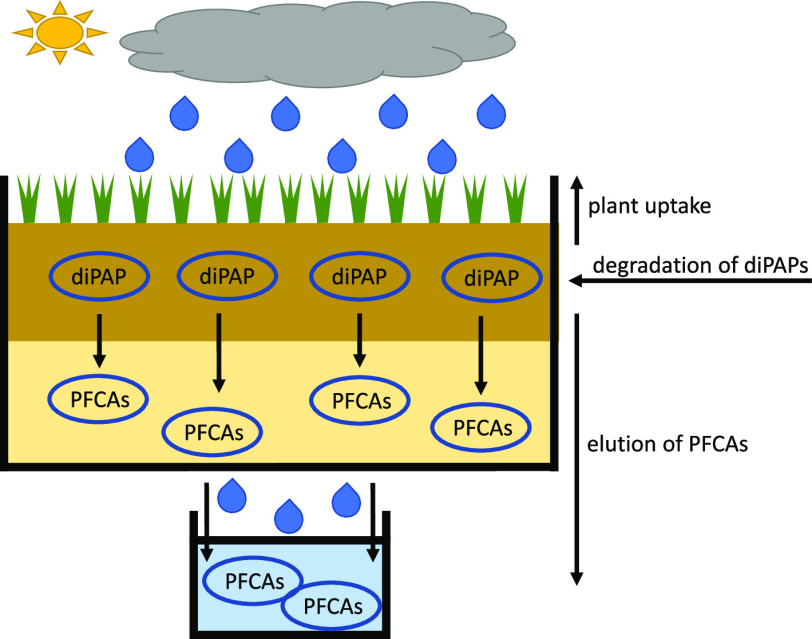

In this study, 6:2 and 8:2 polyfluoroalkyl phosphate diester (diPAP) were individually investigated in lysimeters under near-natural conditions. Leachate was sampled for 2 years, as was the soil after the experiment. In the leachate of the diPAP-spiked soils, perfluorocarboxylic acids (PFCAs) of different chain lengths were detected [23.2% (6:2 diPAP variant) and 20.8% (8:2 diPAP variant) of the initially applied molar amount]. After 2 years, the soils still contained 36–37% 6:2 diPAP and 41–45% 8:2 diPAP, respectively, in addition to smaller amounts of PFCAs (1.5 and 10.6%, respectively). Amounts of PFCAs found in the grass were low (<0.1% in both variants). The recovery rate of both 6:2 diPAP and 8:2 diPAP did not reach 100% (63.9 and 83.2%, respectively). The transformation of immobile diPAPs into persistent mobile PFCAs and their transport into the groundwater shows a pathway for human exposure to hazardous PFCAs through drinking water and irrigation of crops.

## Introduction

Per- and polyfluoroalkyl substances (PFAS) are a large group of anthropogenic chemicals with several thousands of individual substances currently known.^1^ PFAS are used in many industrial sectors, that is, in galvanic processes, as coatings for paper and textile goods, and in fire-fighting foams.^2^ Within this group, perfluoroalkyl acids (PFAAs), including the groups of perfluorocarboxylic acids (PFCAs) and perfluorosulfonic acids (PFSAs),^3^ have been extensively investigated with regard to their environmental and toxicological behavior. Certain PFAAs, such as perfluorooctanoic acid (PFOA, C_8_) and perfluorooctane sulfonic acid (PFOS, C_8_), have been detected ubiquitously in plant,^4^ animal,^5^ and human tissues^6^ as well as in various soil^7^ and water samples^8^ from around the world. They are described as environmentally persistent, bioaccumulative, and toxic substances, which makes them of great concern for human health and environmental safety.^9−11^ These adverse properties resulted in the extensive ban of production and use of both compounds within the European Union.^12^

In addition to PFAAs, various precursor compounds have drawn scientific attention during the last few years. They form a heterogeneous group of fluorine-containing alkyl substances, which can degrade and ultimately transform into PFAAs of different chain lengths by various degradation processes.^13^ Due to their transformation ability, precursor compounds present an additional possible source of PFAAs and thus contribute to the total PFAS burden of humans and the environment.

Several PFAS precursor substances have already been detected in animal and human tissues, that is, *N*-methyl perfluorooctanesulfonamidoacetate and *N*-ethyl perfluorooctane-sulfonamidoacetate in human blood,^14^ fluorotelomer sulfonates, and polyfluoroalkyl phosphates (PAPs) in bream liver^15^ and perfluorooctane sulfonamide in pilot whale muscle tissue.^16^

In contaminated agricultural soils in Southwest Germany, precursors were found to make up a considerable proportion of the total measured PFAS amount,^17^ which underlines the importance of investigating their environmental properties. Among all precursors present in the soil, two disubstituted polyfluoroalkyl phosphates (diPAPs), namely, 6:2 diPAP and 8:2 diPAP, represent a decisive proportion of the total PFAS amount.^15^ The contamination of these soils can be attributed to the application of waste from the paper industry, where diPAPs are used as coating agents for paper and cardboard.^17,18^ DiPAPs have been shown to transform into PFCAs of different chain lengths with two PFCA molecules resulting from the degradation of one diPAP molecule.^19,20^ Further properties regarding environmental behavior have not yet been adequately investigated.

In order to investigate the environmental behavior of PFAS, lysimeter studies were performed under near-natural conditions. In contrast to non-confined field tests, the leachate, the plants growing on the soil, as well as the soil can be analyzed in lysimeter studies. This has the advantage that mass balances and transfer factors can be calculated.^21−24^ The major results of the existing PFAS lysimeter studies were that (i) retardation differs between different PFAS and (ii) the mass balances could not be closed for some PFAS, even though both studies exclusively used non-degradable PFAS. To the best of our knowledge, no field lysimeter studies have been performed applying PFAS precursor substances to the soils, which would provide information about emerging transformation products and their transport behavior in soil.

Thus, we applied two diPAPs (6:2 diPAP and 8:2 diPAP) to field lysimeters and analyzed the leachate, the plants, and the soils for diPAPs and known transformation products for 2 years. The larger aim of this study was to derive information about the contribution of diPAPs to the total PFAS burden in environmental matrices. Furthermore, the fate of diPAP-containing contamination sites in the future may be assessed more accurately using our results.

## Materials and Methods

### Chemicals

Both applied diPAP substances (6:2 diPAP and 8:2 diPAP) were custom-synthetized at the University of Giessen, Germany. A purity of >98% each was determined using phosphor and hydrogen NMR as well as mass spectrometry. Analytical PFAS standards and isotope labeled internal standards (purity >99% each) were obtained from Wellington Laboratories (Guelph, Canada; complete list of standards can be found in Table S1 in the Supporting Information, SI). Sodium carbonate (Na_2_CO_3_ ≥ 99.5%), sodium bicarbonate (NaHCO_3_ ≥ 99.0%), and concentrated ammonia solution (25%) were purchased from Merck (Darmstadt, Germany). Tetrabutylammonium hydrogensulfate (TBA ≥99%) and ammonium acetate for liquid chromatography–mass spectrometry (LC–MS) were from Sigma Aldrich (St. Louis, MO, USA). Methyl *tert*-butyl ether (MTBE ≥99.7%) was from Honeywell (Charlotte, NC, USA). Potassium persulfate (≥99%) and LC–MS grade methanol (MeOH) were obtained from Fisher Scientific (Waltham, MA, USA). Nitrogen gas (grade 5.0) was from Messer (Bad Soden am Taunus, Germany). Concentrated hydrochloric acid (37%), water (LC–MS grade), and sodium hydroxide micro-granules (NaOH ≥99.5%) were from Th. Geyer (Renningen, Germany).

### Setup of the Lysimeters

**Figure 1:**
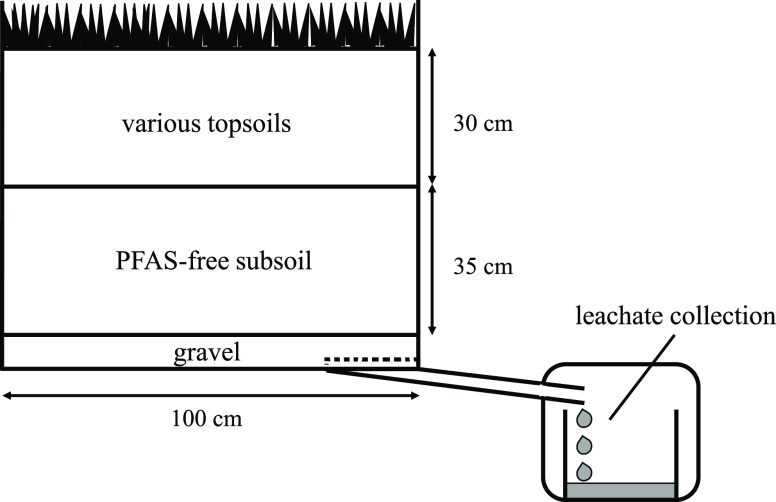
Setup of a study lysimeter.

To investigate the behavior of 6:2 diPAP and 8:2 diPAP in soil, six outdoor lysimeters were set up in Schmallenberg, Germany. The aluminum lysimeters embedded in the soil had dimensions of 100 cm × 100 cm × 80 cm (length × width × height). All lysimeters were first filled with a 5 cm layer of commercially available coarse gravel (tested PFAS-free) to prevent the formation of waterlogging and to improve the drainage of the leachate into the collection vessel. Subsequently, 600 kg fresh matter (FM) of sandy subsoil (RefeSol-01-A from a depth of 30–58 cm) was filled into each lysimeter resulting in a layer height of 35 cm. RefeSol-soil was a standard soil approved by the German Federal Environment Agency (UBA) for test procedures in accordance with the Federal Soil Protection Act, which is characterized in its soil parameters (see Table S2). It was tested PFAS-free prior to the study. The lysimeters only differed with respect to the topsoils used. Figure 1 shows the setup of a lysimeter used in this study.

For topsoil preparation, 4 kg of PFAS-free standard topsoil (RefeSol-01-A from a depth of 0–30 cm, dry matter fraction: 93.06%) was manually sieved to a grain diameter of <2 mm (metal sieve) and was then spiked with one of the two substances, respectively. For the application of 6:2 diPAP, 838 mg of the solid substance was added to 5 mL of MeOH and treated in an ultrasonic bath for 5 min (room temperature) until dissolved. For the application of 8:2 diPAP, a higher volume of MeOH was necessary due to the lower solubility. Thus, 838 mg of solid 8:2 diPAP was dissolved in 100 mL of MeOH using an ultrasonic bath treatment (15 min, 30 °C). The two diPAP solutions were then separately added to 2 kg of the sieved soil each, resulting in two spiked soil pre-mixtures. After the complete evaporation of the MeOH overnight (room temperature) both soil pre-mixtures were individually incorporated into standard RefeSol-01-A topsoil using a cement mixer (1.5 kg of spiked soil pre-mixture plus 336 kg of topsoil). The final mixtures were poured into the lysimeters and were slightly compressed. Two replicates were prepared for each variant. The final diPAP content in the topsoils was 2.00 mg/kg dry matter (DM).

PFAS-free field soils from the sites of Augustenberg and Forchheim, Germany, were used for two additional lysimeters, which served as controls for the possible entry of PFAS via atmospheric pathways (i.e., by precipitation or wind) into the study system. A total mass of 337.5 kg of field soil was placed in each lysimeter, which corresponded to a layer height of 30 cm each after slight compression. Soil characterization is shown in Table S2.

After filling, a commercial grass mixture was sown on all lysimeters to minimize erosion by wind and to avoid crust formation. The system was not artificially irrigated but was subjected only to natural rainfall. Leachate samples were taken variably during the whole experimental duration (April 2019 to April 2021) according to the accumulated leachate volume. While samples could not be taken regularly, for example, during the dry summer months due to high water evaporation resulting in no leachate formation, sampling intervals were shortened (twice per week) during weather periods with high precipitation. Leachate volumes are shown in Figure S1. After a duration of 2 years, soil samples were taken in five depths (height of 12 cm each) per lysimeter with a metal soil sampler. Grass was harvested in November 2019 and November 2020 using a hand mower and was later analyzed for PFAS contamination.

### Analytical Methods

In this study, the sample analysis was based on both a target method with TBA to quantify individual PFAS components as well as a sum parameter method (dTOP assay) to estimate the total PFAS amount in the surveyed experimental compartments (soil, water, and plant). This was necessary to account for any possible degradation intermediates, which may form during the degradation process of the diPAPs. All further information on the analytical parameters, instruments used, and laboratory equipment are shown in Tables S3–S6.

### Target Method

To perform the target method, 1 mL of leachate, 0.5 g of homogenized grass, or 1 g of soil material, respectively, was added to a 15 mL tube (polypropylene, PP). The homogenization of the grass samples was performed by drying the sample to weight constancy in a drying oven (3 days at 40 °C) and a subsequent treatment in a common kitchen blender. As internal standards, 100 μL of a mixture of isotope labeled PFAS standards (100 μg/L each, see Table S1) was added (TBA-IS). After the subsequent addition of 2 mL of a 0.25 M sodium bicarbonate/sodium carbonate buffer solution (hereafter referred to as carbonate buffer), 1 mL of a 0.5 M TBA solution (pH 10) and 5 mL of MTBE, the tube was capped and shaken for 10 min on a shaker (Vortex, 2000 rpm). After treatment of the samples in an ultrasonic bath (10 min, room temperature) and shaking for 10 min (Vortex, 2000 rpm), the samples were centrifuged (4700 rpm, 5 min). The organic supernatant was removed and pipetted into a new 15 mL PP tube. The supernatant was evaporated to dryness under a nitrogen stream at 40 °C, and the residue was subsequently taken up in 1 mL MeOH. After shaking (10 min, Vortex, 2000 rpm) and treatment in an ultrasonic bath (5 min, room temperature), the methanolic solution was transferred to a PP LC vial and measured by liquid chromatography coupled with high-resolution mass spectrometry (LC-HRMS).

### Direct Total Oxidation for Solid Samples

The total oxidizable precursor (TOP) assay, first described by Houtz and Sedlak, includes an oxidation step with potassium persulfate to transform all oxidizable PFAS precursors into PFAAs of different chain lengths, which can then be quantified by basic target methods.^25^ This method is limited to water samples or extracts of solid matrices. In this study, a modification of the TOP assay as described by Göckener et al.^26^ was used. This so-called direct TOP assay (dTOP assay) is an adaption of the TOP assay to solid matrices such as plant or soil samples without any prior extraction steps. This circumvents a potential loss of substances during extraction and thus leads to a more comprehensive overview on the total PFAS burden.

To perform the dTOP assay for soil, 100 mg of sample material was weighed into a 250 mL PP bottle and 100 μL of a mixture of isotope labeled PFAS standards in methanol (only PFCAs and PFSAs, 100 μg/L each, see Table S1) as an internal standard (dTOP-IS) was added. After evaporation of the MeOH under a stream of nitrogen (40 °C), 100 mL of an alkaline potassium persulfate solution (125 mM K_2_S_2_O_8_; 500 mM NaOH) was added, and the bottle was capped. After manual shaking for 30 s, the samples were oxidized for 7 h in a drying oven at 85 °C. After cooling to room temperature, pH was adjusted to pH 6 ± 0.5, which was achieved by adding concentrated hydrochloric acid (HCl) and checked with a pH meter.

Subsequent solid phase extraction (SPE) was performed using SPE cartridges (Oasis WAX, 3 cm^3^, 60 mg, Waters), which have a weak anion exchange material as the active phase. The cartridges were first washed with 5 mL of 0.1% ammonia solution in MeOH followed by 5 mL of MeOH. After equilibration of the cartridge twice with 5 mL of water each time, the sample was applied (1 drop per second, normal pressure). If necessary, a weak vacuum (10 mbar) was applied to increase the run rate to one drop per minute. Following the complete run of the sample, 5 mL of water was used twice to wash the cartridge before it was dried by applying a vacuum (15 mbar) for 5 min. Elution was performed with 5 mL each of MeOH and 0.1% NH_3_ solution in MeOH.

The combined eluates were then evaporated to dryness in a nitrogen stream at 40 °C and taken up in 1 mL MeOH. After shaking (Vortex, 2000 rpm) for 10 min and treatment in an ultrasonic bath (5 min, room temperature), the solution was transferred to a PP LC vial and measured by LC-HRMS.

### Direct Total Oxidation for Liquid Samples

To perform the dTOP assay for leachate samples, 100 μL of the dTOP-IS solution was pipetted into a 15 mL PP tube. The methanolic solution was evaporated to dryness in a nitrogen stream (40 °C). One milliliter of leachate was pipetted into the tube and 1 mL of an alkaline potassium persulfate solution (125 mM K_2_S_2_O_8_; 500 mM NaOH) was added. After sealing, the tube was shaken manually (30 s) and the sample was oxidized in a drying oven at 85 °C for 7 h. After cooling, 3 mL of 0.25 M carbonate buffer, 1 mL of a 0.5 M TBA solution (pH 10), and 5 mL of MTBE were added and the sample was shaken for 10 min (Vortex, 2000 rpm). After treating the samples in an ultrasonic bath (10 min) and another shaking step (10 min, Vortex, 2000 rpm), the samples were centrifuged (4700 rpm, 5 min). The organic supernatant was removed and transferred to a new 15 mL PP tube. The supernatant was evaporated to dryness in a nitrogen stream (40 °C), and the residue was taken up in 1 mL MeOH. After shaking (10 min, Vortex, 2000 rpm) and treatment in an ultrasonic bath (5 min), the methanolic solution was transferred to a PP LC vial and measured by LC-HRMS.

### Dry Matter Determination

To achieve comparability of PFAS content in solid sample matrices, the results obtained were always related to the dry mass of the matrix. To determine the dry matter content, 3 g of each sample was weighed into a halogen dryer (Mettler Halogen Moisture Analyzer HB43-S) and heated to 105 °C in the dryer until weight constancy.

### Evaluation

Thermo XCalibur software (version 3.0.63, Thermo Fisher Scientific) was used to evaluate the measurement data. The quantitative evaluation was based on a calibration series, which was created for all investigated substances combined. For this purpose, a calibration with 10 different concentration levels (0.1, 0.3, 0.5, 0.7, 0.9, 2, 4, 6, 8, and 10 μg/L) was used. The concentrations of the internal standards were 10 μg/L each.

### Validation

For the determination of PFAS content in all samples to be analyzed, both the target method and the sum parameter determination (dTOP assay) were first validated for the different matrices (soil, leachate, and plant). The validation was carried out according to guideline SANTE/12682/2019,^27^ which is applied in the field of registration studies of plant protection products. The analytical methods were assumed valid if the recoveries at the LOQ (limit of quantification) and tenfold LOQ were between 70 and 120% and the standard deviation of 5 samples was less than 20%. This could be achieved for both the target method and the dTOP assay. For the target method, the LOQ of each analyte was 0.5 μg/L leachate or 0.5 μg/kg FM matrix. The LOQ for the dTOP assay was 0.5 μg/L leachate or 5 μg/kg FM matrix after oxidation for all PFCAs and PFSAs tested.

## Results and Discussion

Prior to the start of the experiment, all soils were analyzed for their respective PFAS content by target analysis, before filling them into the lysimeters. As expected, no PFAS concentrations above LOQ were found in the topsoil in either of the control lysimeters. The topsoils in the four lysimeters spiked with 6:2 diPAP and 8:2 diPAP, respectively, showed a diPAP content of about 2 mg/kg DM (6:2 diPAP variant: 1997 ± 308 μg/kg DM; 8:2 diPAP variant: 2057 ± 347 μg/kg DM) after application. Large standard deviations can be explained by the fact that very little substance was applied to several hundred kilograms of soil, making homogeneous incorporation difficult. In addition to the actual applied substance, traces of perfluoropentanoic acid (PFPeA, C_5_) and perfluorohexanoic acid (PFHxA, C_6_) of 1.9–2.2 μg/kg DM were also detected in the soil of the 6:2 diPAP variant and PFOA (2.8 μg/kg DM) in the soil of the 8:2 diPAP variant. The levels, however, were assessed as negligible for the study evaluation.

During the entire study duration of 2 years, no PFAS above LOQ were detected in the leachate of the control lysimeters. Furthermore, both the grass samples and the soil samples taken from the control lysimeters were PFAS free (<LOQ), demonstrating that no significant PFAS entry from the environment into the study system occurred.

### 6:2 diPAP Application

#### Leachate

**Figure 2:**
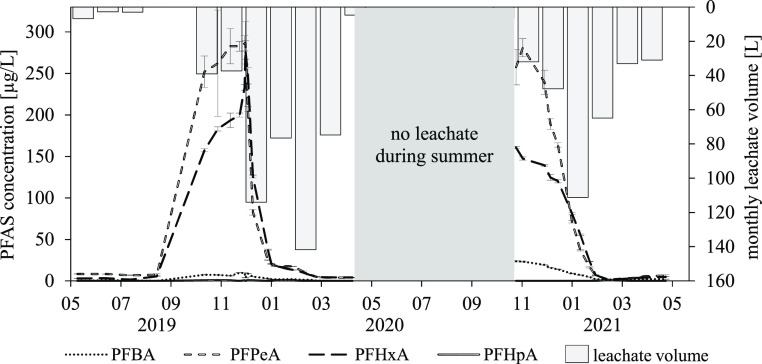
PFAS concentrations in the lysimeter leachate over time and monthly leachate volumes of the 6:2 diPAP application variant; target method; mean value from six analytical replicates; and standard deviation for PFAS concentrations.

In the leachate of the lysimeters treated with 6:2 diPAP, the application substance could not be detected during the 2-year experimental period; however, 4transformation products were found. PFPeA and PFHxA were detected as the main degradation products of 6:2 diPAP, while perfluorobutanoic acid (PFBA, C_4_) and perfluoroheptanoic acid (PFHpA, C_7_) could only be detected at lower concentrations (Figure 2). Because the concentrations in the leachate of both experimental lysimeters developed similarly over time, mean concentrations will be presented.

In the first 4 months of the experiment, only low concentrations (<10 μg/L) of the investigated PFCAs could be detected in the leachate samples. With the onset of autumn 2019 and the resulting higher leachate volumes, the concentrations of PFPeA and PFHxA increased steeply. The concentrations of both PFCAs reached their maxima in December 2019 (study month 8), with a PFPeA concentration of 287 μg/L and a PFHxA concentration of 278 μg/L. This was followed by a steep decrease in the concentrations of both substances to about 4.0 μg/L each in the leachate of the last sampling of the first experimental year (2019/2020). The decrease in concentration indicates that the PFCAs formed from the transformation of the 6:2 diPAP were almost completely removed from the soil system. PFBA was detected at concentrations not exceeding 10 μg/L throughout the first year of the experiment, while PFHpA was quantified only in individual samples at concentrations close to LOQ (0.5 μg/L). No 6:2 diPAP concentrations above the LOQ were detected in the leachate.

From the months of May to the end of September 2020 (study months 13 to 17), no leachate samples could be collected. With the first sampling after the dry period in October, a new increase of PFBA, PFPeA ,and PFHxA concentrations in the leachate could be observed. As in the first half of the experiment, PFPeA (maximum: 281 μg/L) exhibited the highest concentrations among the PFCAs present, followed by PFHxA (maximum: 160 μg/L) and PFBA (maximum: 23.7 μg/L). After reaching maximum concentrations in November or December 2020 (study months 19 to 20), the concentration curves for all three substances again dropped rapidly, so that concentrations in February 2021 (study month 22) were below 10 μg/L each. PFPeA and PFHxA were identified as the main degradation products of 6:2 diPAP in several studies regarding different matrices and degradation paths.^19,20,28^ The new increase in concentrations after several months of no leachate (May to September 2020) indicates enhanced 6:2 diPAP transformation during the summer, as well as subsequent discharge of the resulting PFCAs as transformation products from the lysimeter with the leachate. This can possibly be attributed to the higher temperatures in the soil system and the associated increased microbiological activity. The activity of microorganisms was identified in studies by Lee et al.^19^ as essential for the degradation of monoPAPs of different chain lengths and 6:2 diPAP in sewage sludge. Under sterile experimental conditions, however, no 6:2 diPAP degradation could be detected by the authors within an experimental period of 92 days.^19^ Consequently, the influence of non-microbial degradation pathways on 6:2 diPAP degradation can be assumed to be low, at least for the first degradation step. The subsequent discharge of the formed PFCAs with the leachate occurred in the autumn months due to the higher precipitation rate.

Overall, the detection of PFPeA and PFHxA as main degradation products of 6:2 diPAP is in line with the findings of other studies with different study setups (Table 1). In addition to the formation of PFCAs of different chain lengths, several studies also identified fluorotelomer carboxylic acids (FTCAs), fluorotelomer unsaturated carboxylic acids (FTUCAs), and fluorotelomer alcohols (FTOHs) as degradation products of 6:2 diPAP. These substances were not assessed in the present study.

**Table 1:** Overview of Degradation Products of 6:2 diPAP and 8:2 diPAP Observed in the Present and Other Studies

substance	study type	measured degradation products	reference
6:2 diPAP	lysimeters	PFPeA > PFHxA > PFBA > PFHpA	present study
	unsaturated soil columns	PFPeA > PFHxA > PFBA > PFHpA	Weidemann et al.^29^
	soil-plant system	PFPeA > PFHxA > PFBA > PFHpA	Just et al.^30^
	soil-plant system	PFPeA > PFHxA > PFBA	Scheurer et al.^31^
	soil-plant system	major: PFHxA, minor: PFBA, PFPeA, PFHpA, 6:2 FTCA, 6:2 FTUCA, 5:3 FTCA, 5:3 FTUCA	Lee et al.^19^
	aerobic microbial incubation	PFPeA, PFHxA, PFHpA, 6:2 monoPAP, 6:2 FTOH, 6:2 FTCA, 6:2 FTUCA, 5:3 FTCA	Lee et al.^32^
	aerobic soil in dynamic reactors	5:3 FTCA > PFPeA > PFHxA, minor: PFBA, 5:2 sFTOH	Liu and Liu^20^
8:2 diPAP	lysimeters	PFOA > PFHpA > PFHxA > PFPeA > PFBA	present study
	unsaturated soil columns	PFOA > PFHpA > PFHxA > PFPeA > PFBA	Weidemann et al.^29^
	soil-plant system	PFOA > PFHpA > PFHxA > PFPeA > PFBA	Just et al.^30^
	compost-amended soil	major: PFOA, minor: 8:2 monoPAP, 8:2 FTUCA, 8:2 FTCA, 7:3 FTCA, PFBA, PFPeA, PFHxA, PFHpA, PFNA	Bizkarguenaga et al.^33^
	aerobic soil in dynamic reactors	major: PFOA, minor: PFHpA, PFHxA, 7:3 FTCA, 7:2 sFTOH	Liu and Liu^20^

Examination of the leachates by the dTOP assay resulted in consistently comparable PFCA concentrations compared to the target analysis (see Figure S2), ruling out a significant presence of oxidizable PFAS precursors, particularly 6:2 diPAP in this variant.

#### Soil

**Figure 3:**
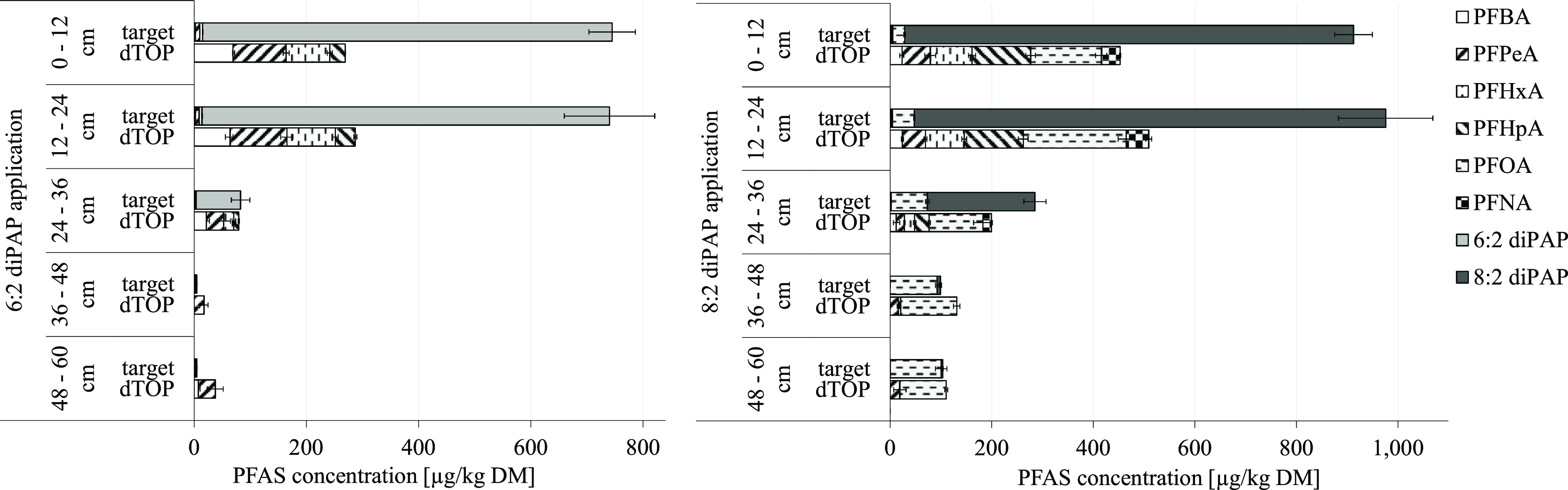
PFAS content in five depths of the lysimeter soil of the 6:2 diPAP application variant and the 8:2 diPAP application variant after the end of the 2 year of experiment; target method (six analytical replicates); and sum parameter determination (dTOP assay, four analytical replicates) with the indication of the standard deviation.

To ensure a complete balance, the lysimeter soils were also analyzed for PFAS in addition to the leachate. In the soil of the 6:2 diPAP lysimeters, high levels of the applied substance were detected in the upper two soil layers (0–24 cm) after the end of the test period of 2 years (Figure 3).

Consequently, degradation of the 6:2 diPAP was not completed after the experimental period of 2 years with a respective content of 726 and 730 μg/kg DM (36–37% of the initial concentration) in the topsoil layers (0–12 cm and 12–24 cm) of the 6:2 diPAP variant. This underlines the possibility of a long-term substance degradation and the subsequent formation of PFCAs from the finite diPAP reservoir, which is considered as critical for the environment.

In the lower soil layers, 6:2 diPAP could only be detected at low levels (<80 μg/kg DM), which argues against a substantial substance migration through the soil horizon. This distribution may be the result of unintentional mixing of the PFAS-free subsoil and the 6:2 diPAP-containing study soil during the installation of the lysimeters or during soil sampling after the study time of 2 years. Therefore, 6:2 diPAP can be assumed to be a strongly immobile substance in soil. PFCAs (C_4_ to C_6_) were quantified in all soil layers with a content below 7.6 μg/kg DM each, which, in contrast to the behavior of 6:2 diPAP, indicates an almost complete leaching of these short-chain substances. This result is comparable to other PFAS lysimeter studies, where a total leaching of short-chain PFCAs and a retardation of long-chain PFCAs was observed.^22,23^ The results at the end of the experiment in late spring support the assumption that the transformation of diPAPs mainly takes place at higher temperatures, that is, in summer. Assuming a first-order kinetic degradation model, a dissipation time of 50% of the applied diPAP mass (DT_50_) was calculated based solely on the start and end concentrations of 6:2 diPAP in the soil (see eq S1). These calculations result in a DT_50_ of 507 days for the dissipation of 6:2 diPAP in the soil. In a study by Liu and Liu,^20^ a DT_50_ of 12 days was calculated for 6:2 diPAP in aerobic soil, but the study setup differed heavily as a semi-dynamic reactor approach was chosen. Accordingly, the influence of environmental parameters in the test system (temperature, soil moisture, etc.) on diPAP degradation over time was not considered. In a study by Just et al.,^30^ a DT_50_ of 33 days was calculated for the degradation of 6:2 diPAP in a soil-plant system. Here, maize plants were cultivated on 6:2 diPAP-applied soil in Mitscherlich pots under semi-outdoor conditions, clearly limiting the comparability of the different DT_50_ values.

After oxidation by dTOP assay, 6:2 diPAP could no longer be found, but the reaction products PFBA, PFPeA, and PFHxA and smaller amounts of PFHpA were detected. In the uppermost soil layer (0–12 cm), the sum of the four PFCAs was 270 μg/kg DM, in the soil layer below (12–24 cm) 290 μg/kg DM. Both values were clearly below the previously determined 6:2 diPAP content. This mass loss during the oxidation of diPAPs was previously observed by Göckener, et al.^26^ and can be explained by the oxidation of the central phosphate group and the non-fluorinated carbon atoms within the diPAP molecule as well as by a possible formation of ultra-short-chain PFAAs, such as trifluoroacetic acid (TFA, C_2_) and perfluoropropanoic acid (PFPrA, C_3_) during the dTOP assay.^34^ Due to the small amount of PFAS detected via the dTOP assay compared to the target method, the presence of unknown precursor compounds can be assumed to be negligible.

#### Grass

In addition to the leachate and the soil, the grass cover on the lysimeters was also examined for PFAS (see Table S7). In the grass grown on the lysimeter with the 6:2 diPAP application, PFBA, PFPeA, and PFHxA could be detected in both vegetation periods with a total content of 7750 μg/kg DM (2019) and 7260 μg/kg DM (2020), respectively. It must be noted, however, that the grass matrix only contributed to a small proportion of the total system mass (<130 g DM) compared to the leachate and soil. Hence, the high PFAS concentrations in the plant make up less than 0.1% of the overall detected PFAS mass and consequently only have a small influence on the total PFAS balance. A similar result was found by Just et al.^30^ for the uptake of 6:2 diPAP degradation products into maize plants with a recovery rate of 1.4% of the initially applied PFAS amount in the aboveground plant compartments.

In both years, PFPeA represented the main component with about 80% of the total PFCA content in the grass sample. This corresponds to the fact that PFPeA was already identified in the leachate as the main product from the substance degradation of the 6:2 diPAP. The fact that the PFBA content was higher in the vegetation than the PFHxA content, which was detected more frequently in the leachate, suggests a chain length dependence on the substance uptake into the plant. This dependence has been described for different plants and study configurations, and it was attributed to the higher water solubility of short-chain PFCAs, which then caused a higher substance uptake into plants via water uptake.^35,36^ In addition to PFCAs, low levels of 6:2 diPAP were present in the grass with 24.0 μg/kg DM in crop year 2019 and 35.2 μg/kg DM in the following year. The low 6:2 diPAP levels compared to PFCA levels can be explained either by surface contamination of the plant samples by soil particles or by an uptake of the application substance into the plants. Atmospheric deposition is assumed to be negligible, as no PFAS were detected in the system compartments (soil, leachate, and grass) of the control variant. Studies by Bizkarguenaga et al.^33^ and Just et al.^30^ demonstrated evidence of minimal diPAP uptake into plants.

#### Mass Balance

A mass balance of the PFAS loads in the overall system was carried out in order to obtain a comprehensive understanding of the study results. For the conversion from diPAPs to PFCAs, the balance was calculated on a molar basis. It should be noted here that 2 mol of PFCAs could theoretically be formed from 1 mol of 6:2 diPAP based on the molecular structure. Therefore, a conversion factor of 2 had to be applied when calculating the molar recovery rates for PFCAs (RR_PFCA_). To calculate the RR_PFCA_, the substance amounts of all detected PFCAs in the three surveyed matrices (leachate, soil, and grass) were individually considered in relation to the amount of diPAP applied at the beginning of the experiment (see eq S2). A separate determination for the recovery rates of the diPAPs (RR_diPAP_) without any conversion factor was performed (see eq S3). The RR_PFCA_ for the leachate of the 6:2 diPAP variant was 23.2%. Thus, about one-fifth of the theoretical maximum amount of material has been discharged with the leachate from the lysimeter in the form of various PFCAs. The PFCAs detected in the soil account for only a small proportion of the total balance (1.5%), as does the proportion of PFAS in the grass cover (<0.1%). Based on the 6:2 diPAP content determined at the end of the experiment in the soil layers and the mass of the soil layers, 39.2% of the initial amount of substance was recovered in the soil. Consequently, a total of 63.9% of 6:2 diPAP applied to the system at the beginning of the experiment was found in the three matrices investigated (leachate, soil, and grass) at end of the study. This shortfall was already observed in a study by Weidemann et al.^29^ with unsaturated soil columns. They calculated a similar total recovery rate of 49.0% for 6:2 diPAP after a study duration of 105 weeks. Differences can be explained by the different study setups, especially the constant watering and temperature in the soil column study in contrast to the irregular natural rainfall and temperature fluctuations in our lysimeter study.

The incomplete substance recovery may have various cases: First, the analytical method used in this work was not capable of quantifying ultra-short-chain PFCAs (TFA and PFPrA). The ubiquitous occurrence of these substances has been described in several studies, as has their large contribution to the total PFAS load of various samples studied.^37,38^ Because PFCAs are a homologous series, formation from 6:2 diPAP analogous to PFCAs with chain lengths < C_4_ via biodegradation processes is conceivable for TFA and PFPrA; however, no evidence is available to suggest this. Furthermore, the volatility of the substances must also be taken into account. For example, TFA is considered a “volatile organic compound” (VOC) according to EU Directive 2010/75/EU^39^ and would thus be able to leave the soil system by volatilization during the test period. Strong interactions between the analytes and soil constituents that cannot be resolved by the analytical methods used (target analysis and dTOP assay) must be considered. This is generally referred to as the formation of non-extractable residues (NERs) and has been described in other PFAS studies with soil.^23,40^ Only a total fluorine determination by digestion, not carried out in this work, would include NER in the mass balance, but this would also result in a loss of any structural information. In addition to the formation of NER, an accumulation of PFAS on surfaces of the test system, in particular on the walls of the lysimeter, cannot be excluded.

### 8:2 diPAP Application

#### Leachate

**Figure 4:**
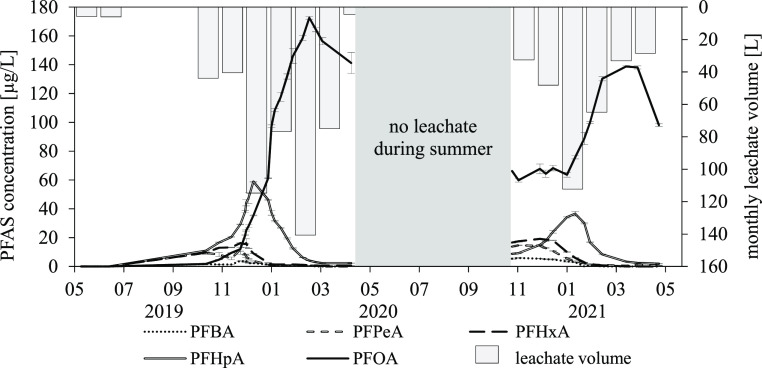
PFAS concentrations in the lysimeter leachate over time and monthly leachate volumes of the 8:2 diPAP application variant; target method; mean value from six analytical replicates; and standard deviation for PFAS concentrations.

In the leachates of the lysimeter treated with 8:2 diPAP, no 8:2 diPAP but five PFCAs with chain lengths from C_4_ to C_8_ could be detected as transformation products. The main degradation product observed was PFOA, which shows the highest concentrations in the water samples during the experimental period (Figure 4).

PFOA reached a maximum concentration of 173 μg/L (February 2020, study month 10), followed by PFHpA with 58.9 μg/L. PFHxA, PFPeA, and PFBA were always observed at concentrations less than 20 μg/L. The concentration curves of the PFCAs showed a chain length dependence of the elution period. For example, the short-chain PFCAs (C_4_ to C_6_) reached their maximum concentration as early as November 2019 (study month 7), whereas for PFHpA this concentration was not reached until December 2019 (study month 8) and for PFOA until February 2020 (study month 10). Considering a concurrent formation of all PFCAs during the degradation process, this can be explained by a stronger retardation in the soil with increasing chain length of the acids, as described in the literature for PFAA applications.^23^

In contrast to the 6:2 diPAP variant, not all PFCA concentrations in the 8:2 diPAP variant leachate decreased to near LOQ by spring 2020. Although the concentration of PFOA decreased slowly after reaching the maximum, 141 μg/L PFOA could still be detected in the last water sample of the first year of the experiment. This can be explained by the low water solubility of PFOA and high retardation in the soil compared to shorter chain PFCAs, which results in a reduced ability to be washed out with the leachate.^23,41^

As in the lysimeters treated with 6:2 diPAP, a new increase of the concentrations of all detected PFCAs (C_4_ to C_8_) was also evident in the leachate of the soils treated with 8:2 diPAP in the second year of the experiment. PFOA continued to represent the main substance in all leachate samples with a maximum concentration of 139 μg/L in March 2021 (study month 23). The concentration maximum in the second year of the experiment was thus below that in the first year of the experiment (173 μg/L), indicating a degradation of 8:2 diPAP during the summer and a discharge of PFCAs with the leachate. All other PFCAs reached their concentration maxima in the second year in the months from November 2020 to January 2021 (5.9–36.7 μg/L, study months 19 to 21). As in the case of 6:2 diPAP, concentrations dropped rapidly after reaching the maximum.

The detection of PFOA as the main degradation product of 8:2 diPAP and a minor formation of short-chain PFCAs (C_4_ to C_7_) mainly complies with the results of other studies (Table 1). In addition to the PFCAs investigated in the present study, Liu and Liu^20^ detected 7:3 FTCA and 7:2 sFTOH. These substances were not investigated in the present study. Bizkarguenaga et al.^33^ detected 8:2 monoPAP and polyfluorinated carboxylic acids as intermediates of the 8:2 diPAP degradation as well as PFNA as a final degradation product. In contrast to their study, PFNA could not be detected as a degradation product in our study. However, PFNA was only formed in minimal amounts compared to the PFCAs of chain lengths C_4_ to C_8_ (PFOA > PFHpA > PFHxA > PFPeA > PFBA > PFNA). It is therefore conceivable for the soil system investigated in the present work that PFNA formation from 8:2 diPAP took place to an extent too small to be detectable with the analytical methods used.

Examination of the leachates by dTOP assay (see Figure S3) revealed comparable PFAA concentrations, ruling out the presence of oxidizable PFAS precursors or degradation intermediates.

#### Soil

In the soil of the 8:2 diPAP lysimeter, high levels of the originally applied substance were detected in the upper two soil layers (0–24 cm) (Figure 3), with 848 to 928 μg/kg DM (41–45% of the initial concentration). In the lower soil layers (36–60 cm), 8:2 diPAP was detected at substantially lower levels (<6.4 μg/kg DM). PFCAs (C_5_ to C_7_) were detected at levels below 2.3 μg/kg DM each. As for 6:2 diPAP, 8:2 diPAP can be assumed to be a highly immobile substance in soil systems without any substantial transport into deeper soil layers. Only PFOA as the longest detected PFCA showed elevated levels (23.7–99.9 μg/kg DM) in all soil layers due to its high migration retardation in the soil.^42^ Compared to the diPAP content in the soil of the 6:2 diPAP variant, it is noticeable that the 8:2 diPAP content at the end of the experiment was higher than the 6:2 diPAP content despite the same application concentration (2 mg/kg DM).

A DT_50_ of 677 days was calculated for the degradation of 8:2 diPAP in the soil. In a study by Liu and Liu,^20^ a DT_50_ of more than 1000 days was determined, but the studies are not quite comparable due to the different study setup (semi-dynamic reactor approach in the latter study). The high DT_50_ of 677 days indicates a slower degradation rate of the 8:2 diPAP in the soil system compared to 6:2 diPAP (507 days), which can be attributed to the longer polyfluorinated alkyl side chains, the higher molar weight, and an overall lower microbial bioavailability.^20^ The influence of microbial life on the degradation of diPAPs was described as essential in several studies.^32,43,44^

#### Grass

In the grass cover of the 8:2 diPAP lysimeter, all PFCAs of chain lengths from C_4_ to C_8_ could be detected with PFBA and PFPeA representing the highest proportion (see Table S7). In addition to the PFCAs, 8:2 diPAP could also be quantified in the samples at low levels, although this cannot necessarily be explained by the uptake of the substance into the plant but may also be the result of surface attachment of soil particles to the grass. As no PFAS were detected above LOQ in the control systems, atmospheric substance deposition is expected to be negligible. The total PFAS concentrations in the grass samples were 711 μg/kg DM in crop year 2019 and 596 μg/kg DM in crop year 2020.

#### Mass Balance

The calculation of the RR_PFCA_ in the leachate of the 8:2 diPAP variant amounted to 20.8% and is thus approximately as high as that in the leachate of the 6:2 diPAP variant. After the experimental period of 2 years, 51.8% of the originally applied diPAP substance quantity was still present in the lysimeter soil and thus substantially more than in the 6:2 diPAP variant. The fact that PFCAs in the soil accounted for 10.6% of the total balance is mainly due to the formation of PFOA from 8:2 diPAP, migration of which was more strongly retarded in the soil due to its chain length and thus could not be completely discharged by the end of the experiment (Figure 3). Grass cover accounted for only a minimal portion of the total balance (<0.1%). Overall, 83.2% of the applied quantity of the substance could be recovered in all combined compartments of the soil system. As for the 6:2 diPAP variant, the calculated recovery rate of 8:2 diPAP is higher than the rate Weidemann et al.^29^ calculated in their soil column study (68.4%). As both studies differ in their setups, especially in the water and temperature regularity, the rates cannot be compared directly.

The gap in the recovery rate in both study variants can be explained by evaporation of volatile degradation products, formation of NER within the lysimeter system or an analytical method not capable of detecting ultra-short chain PFAS or intermediates of the degradation process. Accordingly, it is advised to implement studies with special attention to those problems, that is, by performing air trapping above the test system, by complete digestion and the quantification of the total fluorine or by different analytical methods (e.g., gas chromatography methods). Furthermore, the present study underlines the ability of diPAPs to contribute to the total PFAS entry into ground water. As ground water is used as a source of drinking water and for crop irrigation, this poses a risk to human and animal health. It must be pointed out, however, that diPAPs only make up a small proportion of the total number of known PFAS precursors. Consequently, further studies will need to be carried out to take more precursor substances into consideration when discussing the environmental behavior of PFAS.

## Supporting Information Available

Instrumental parameters of the PFAS analysis, properties of the investigated soils, PFAS concentrations in the grass, measured leachate volumes, results of the dTOP assay for the leachate, and all used mathematical equations (PDF)

## Abbreviations

PFAS: per- and polyfluoroalkyl substances
diPAP: polyfluoroalkyl phosphate diester
PFCA: perfluorocarboxylic acid
PFAA: perfluoroalkyl acid
PFSA: perfluorosulfonic acid
PFOA: perfluorooctanoic acid
PFOS: perfluorooctane sulfonic acid
FOSA: perfluorooctane sulfonamide
MTBE: methyl *tert*-butyl ether
MeOH: methanol
FM: fresh matter
DM: dry matter
UBA: German Federal Environment Agency
dTOP assay: direct total oxidizable precursor assay
PP: polypropylene
IS: internal standard
LC-HRMS: liquid chromatography coupled with high-resolution mass spectrometry
SPE: solid phase extraction
LOQ: limit of quantification
PFPeA: perfluoropentanoic acid
PFHxA: perfluorohexanoic acid
PFHpA: perfluoroheptanoic acid
PFBA: perfluorobutanoic acid
FTCA: fluorotelomer carboxylic acid
FTUCA: fluorotelomer unsaturated carboxylic acid
FTOH: fluorotelomer alcohol
DT_50_: dissipation time of 50% of the applied mass
TFA: trifluoroacetic acid
PFPrA: perfluoropropanoic acid
RR: recovery rate
VOC: volatile organic compound
NER: non-extractable residues
