# Supplementary material for: Evaluation of the Transformation and Leaching Behavior of Two Polyfluoroalkyl Phosphate Diesters in a Field Lysimeter Study
Source: J Agric Food Chem. 2022 Nov 2;70(45):14329–38. doi: 10.1021/acs.jafc.2c03334 (PMC9673155; doi:10.1021/acs.jafc.2c03334)
Supplement: Supplementary file 1 — jf2c03334_si_001.pdf [file jf2c03334_si_001.pdf]

Supporting Information for

Evaluation of the Transformation and Leaching Behavior of Two Polyfluoroalkyl Phosphate Diesters (diPAPs) in a Field Lysimeter Study

René Lämmer[1], Eva Weidemann[2], Bernd Göckener[1], Thorsten Stahl[3], Jörn Breuer[4], Janine Kowalczyk[5], Hildegard Just[5], Runa S. Boeddinghaus[4], Matthias Gassmann[2], Hans-Willi Kling[6], Mark Bücking[1,7]

[1] Department of Environmental and Food Analysis, Fraunhofer Institute for Molecular Biology and Applied Ecology IME, Auf dem Aberg 1, 57392 Schmallenberg, Germany

[2]Department of Hydrology and Substance Balance, University of Kassel, Kurt-Wolters-Str. 3, 34125 Kassel, Germany

[3]Chemical and Veterinary Analytical Institute Münsterland-Emscher-Lippe, Joseph-König-Str. 40, 48147 Münster, Germany

[4]Center for Agricultural Technology Augustenberg (LTZ), Neßlerstraße 25, 76227 Karlsruhe, Germany

[5]German Federal Institute for Risk Assessment, Max-Dohrn-Str. 8-10, 10589 Berlin, Germany

[6]Department of Chemistry and Biology, University of Wuppertal, Gaußstraße 20, 42119 Wuppertal, Germany

[7]School of Clinical Sciences at Monash Health, Faculty of Medicine, Nursing and Health Sciences, Monash University VIC 3800, Australia

Corresponding author: rene.laemmer@ime.fraunhofer.de

List of Tables

Table S1: Analytical standards and their isotope labelled internal standard equivalents (Purchased from: Wellington Laboratories, Guelph, Canada). 3
Table S2: Properties of the soil used in the lysimeter experiment. 3
Table S3: Method parameters of the LC system. 4
Table S4: Method parameters of the HRMS system. 4
Table S5: Laboratory products. 5
Table S6: Laboratory equipment. 5
Table S7: PFAS concentrations in the grass cover of the lysimeter variants "6:2 diPAP" and "8:2 diPAP" for both harvest years; target method; mean value from 6 replicates and standard deviation, LOQ of 0.5 µg/kg. 6

List of Figures

Figure S1: Cumulated leachate volumes of the lysimeter experiment during the study time of two years for the 6:2 diPAP variant and the 8:2 diPAP variant. 6
Figure S2: PFAS concentrations in the lysimeter leachate over time and monthly leachate volumes of the 6:2 diPAP variant; dTOP assay; mean value from 6 analytical replicates and standard deviation for PFAS concentrations. 7
Figure S3: PFAS concentrations in the lysimeter leachate over time and monthly leachate volumes of the 8:2 diPAP variant; dTOP assay; mean value from 6 analytical replicates and standard deviation for PFAS concentrations. 7

List of Equations

Equation S1: Calculation of the dissipation time of 50% (DT_{50}) assuming a first order kinetic model using the total experimental time (t), the total diPAP mass in the soil at the beginning of the study (m_0) and at the end of the study (m_t). 8
Equation S2: Calculation of the recovery rate of PFCAs (RR_{PFCA}) from the degradation of diPAPs using the total molar substance amount (n) of diPAPs at the beginning of the study ($diPAPs_{start}$) and of PFCAs at the end of the study ($PFCAs_{end}$). 8
Equation S3: Calculation of the recovery rate of diPAPs (RR_{diPAP}) using the total molar substance amount (n) of diPAPs at the beginning of the study ($diPAPs_{start}$) and of PFCAs at the end of the study ($diPAPs_{end}$).... 8

Table S1: Analytical standards and their isotope labelled internal standard equivalents (Purchased from: Wellington Laboratories, Guelph, Canada).

PFAS class	Analytical standard	Concentration [mg/L]	Internal standard used
PFCAs	PFBA	PFCA mix (2 mg/L per analyte)	M-PFBA
	PFPeA		M-PFPeA
	PFHxA		M-PFHxA
	PFHpA		M-PFHpA
	PFOA		M-PFOA
	PFNA		M-PFNA
	PFDA		M-PFDA
	PFUnDA		M-PFUnDA
	PFDoDA		M-PFDoDA
	7H-PFHpA	50	M-PFHpA
PFSAs	PFBS	PFSA mix (2 mg/L per analyte)	M-PFBS
	PFHxS		M-PFHxS
	PFHpS		M-PFOS
	PFOS		M-PFOS
	PFDS		M-PFOS
PAPs	6:2 diPAP	50	M-6:2 diPAP
	6:2/8:2 diPAP	50	M-8:2 diPAP
	8:2 diPAP	50	M-8:2 diPAP
FTSs	4:2 FTS	50	M-6:2 FTS
	6:2 FTS	50	M-6:2 FTS
	8:2 FTS	50	M-6:2 FTS
FOSAs	FOSAA	50	d3-N-MeFOSAA
	N-MeFOSAA	50	d3-N-MeFOSAA
	N-EtFOSAA	50	d3-N-MeFOSAA
	FOSA	50	d-N-MeFOSA
	N-MeFOSA	50	d-N-MeFOSA
	N-EtFOSA	50	d-N-MeFOSA
Ether based PFAS	HFPO-DA (GenX)	50	M-HFPO-DA
	ADONA	50	M-PFOA
	9Cl-PF3ONS	50	M-PFOS
	11Cl-PF3OUdS	50	M-PFOS
Cyclical PFAS	PFECHS	50	M-PFOS

Table S2: Properties of the soil used in the lysimeter experiment.

Soil	pH [-]	Organic carbon [%]	Clay (< 2 µm) [%]	Silt (2 - 63 µm) [%]	Sand (63 µm - 2 mm) [%]
Augustenberg	7.0	0.8	19.5	45.4	35.1
Forchheim	5.4	1.7	7.7	16.5	75.8
RefeSol 01_A, depth: 0 – 30 cm	5.6	1.1	6.1	17.2	73.1
RefeSol 01_A, depth: 30 – 58 cm	4.9	0.2	5.5	4.5	90.0

Table S3: Method parameters of the LC system.

<table>
<tr><td rowspan="3">HPLC instruments</td><td colspan="4">Acquity nanoUPLC, Waters</td></tr>
<tr><td colspan="4">Acquity nano Binary Solvent Manager</td></tr>
<tr><td colspan="4">Acquity nano Sample Manager, Column Heater</td></tr>
<tr><td>HRMS instrument</td><td colspan="4">Q-Exactive-Plus, Thermo Fisher Scientific</td></tr>
<tr><td>Column</td><td colspan="4">Waters Acquity UPLC® BEH C18, 1.7 µm, 2.1 x 100 mm</td></tr>
<tr><td>Sample temperature</td><td colspan="4">15 °C</td></tr>
<tr><td>Column temperature</td><td colspan="4">55 °C</td></tr>
<tr><td>Flow rate</td><td colspan="4">0.3 mL/min</td></tr>
<tr><td>Injection volume</td><td colspan="4">10 µL</td></tr>
<tr><td>Mobile phase</td><td colspan="4">A: Water/methanol (95/5, v/v) with 2 mM ammonium acetate
B: Methanol with 2 mM ammonium acetate</td></tr>
<tr><td rowspan="5">Gradient</td><td>Time [min]</td><td>Mobile phase A [%]</td><td>Mobile phase B [%]</td><td>Gradient</td></tr>
<tr><td>0</td><td>100</td><td>0</td><td>Initial</td></tr>
<tr><td>10</td><td>0</td><td>100</td><td>6</td></tr>
<tr><td>12</td><td>0</td><td>100</td><td>6</td></tr>
<tr><td>20</td><td>100</td><td>0</td><td>1</td></tr>
</table>

Table S4: Method parameters of the HRMS system.

Scan type	Full-Scan and AIF
Scan range	100 - 1300 m/z
Resolution	70,000 (Full-Scan), 35,000 (AIF)
Polarity	negative
Microscans	1
AGC target [ions]	$3x10^6$
Maximum inject time [ms]	50
Sheath gas flow rate [*]	35
Aux gas flow rate [*]	10
Sweep gas flow rate [*]	2
Spray voltage [|kV|]	3.0
Capillary temperature [°C]	320
S-lens RF level	55.0
Aux gas heater temperature [°C]	350

[*] = arbitrary units

Table S5: Laboratory products.

**Product**	**Manufacturer**
Cellstar® Tubes 15 mL PP	Greiner bio-one, Kremsmünster, Austria
Pasteur pipette 150 mm	Th. Geyer, Renningen, Germany
Pipette tips 1 mL	Greiner bio-one, Kremsmünster, Austria
Pipette tips 200 µL TipOne®	Starlab, Hamburg, Germany
Pipette tips 5 mL Diamond®	Gilson, Middleton, USA
PP screw caps with septum	ThermoFisher Scientific, Waltham, USA
PP vials 2 mL	Agilent, Santa Clara, USA
PP flasks 250 mL	Nalgene, Rochester, USA
PP tube 50 mL	Sarstedt, Nümbrecht, Germany
WAX 6 cc cartridges 150 mg 30 µm	Waters, Milford, USA

Table S6: Laboratory equipment.

**Equipment**	**Model**	**Manufacturer**
Pipettes 100 µL, 1 mL and 5 mL	Research Plus	Eppendorf, Hamburg, Germany
pH meter	Toledo MP220	Mettler, Columbus, USA
Shaker	VORTEX 4 basic	IKA, Staufen, Germany
Nitrogen evaporator	Zymark TurboVap® LC Evaporator	ThermoFisher Scientific, Waltham, USA
Drying oven	kelvitron® t	Heraeus, Hanau, Germany
Ultrasonic bath	Sonorex RK 514 Transistor	Bandelin, Berlin, Germany
Scale		Sartorius, Göttingen, Germany
Centrifuge	Megastar 1.6 R	VWR, Radnor, USA

Table S7: PFAS concentrations in the grass cover of the lysimeter variants "6:2 diPAP" and "8:2 diPAP" for both harvest years; target method; mean value from 6 replicates and standard deviation, LOQ of 0.5 µg/kg.

	**6:2 diPAP [µg/kg DM]**		**8:2 diPAP [µg/kg DM]**	
**compound**	**2019**	**2020**	**2019**	**2020**
PFBA	1,020 ± 80	1,190 ± 210	285 ± 14	276 ± 26
PFPeA	6,350 ± 690	5,580 ± 390	239 ± 20	134 ± 21
PFHxA	382 ± 30	488 ± 118	30.1 ± 2.0	28.3 ± 2.8
PFHpA	< LOQ	1.7 ± 1.0	26.8 ± 1.2	27.2 ± 2.5
PFOA	< LOQ	< LOQ	94.7 ± 7.0	95.3 ± 14.5
6:2 diPAP	24.0 ± 2.0	35.2 ± 6.1	< LOQ	< LOQ
8:2 diPAP	< LOQ	< LOQ	35.0 ± 5.1	35.2 ± 3.5

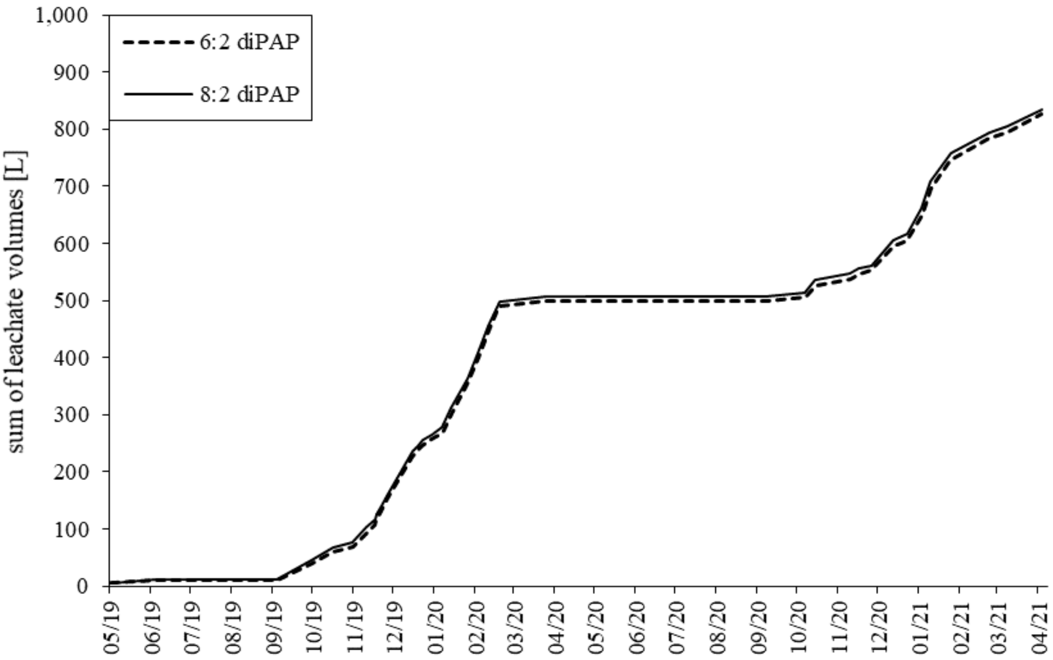

Figure S1: Cumulated leachate volumes of the lysimeter experiment during the study time of two years for the 6:2 diPAP variant and the 8:2 diPAP variant.

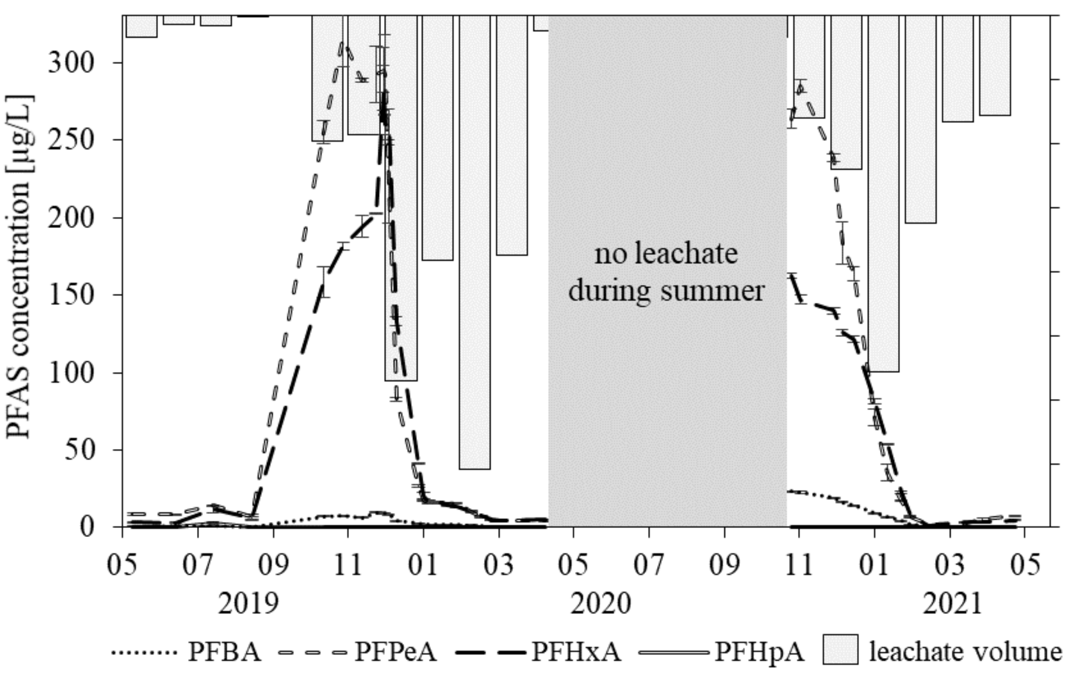

Figure S2: PFAS concentrations in the lysimeter leachate over time and monthly leachate volumes of the 6:2 diPAP variant; dTOP assay; mean value from 6 analytical replicates and standard deviation for PFAS concentrations.

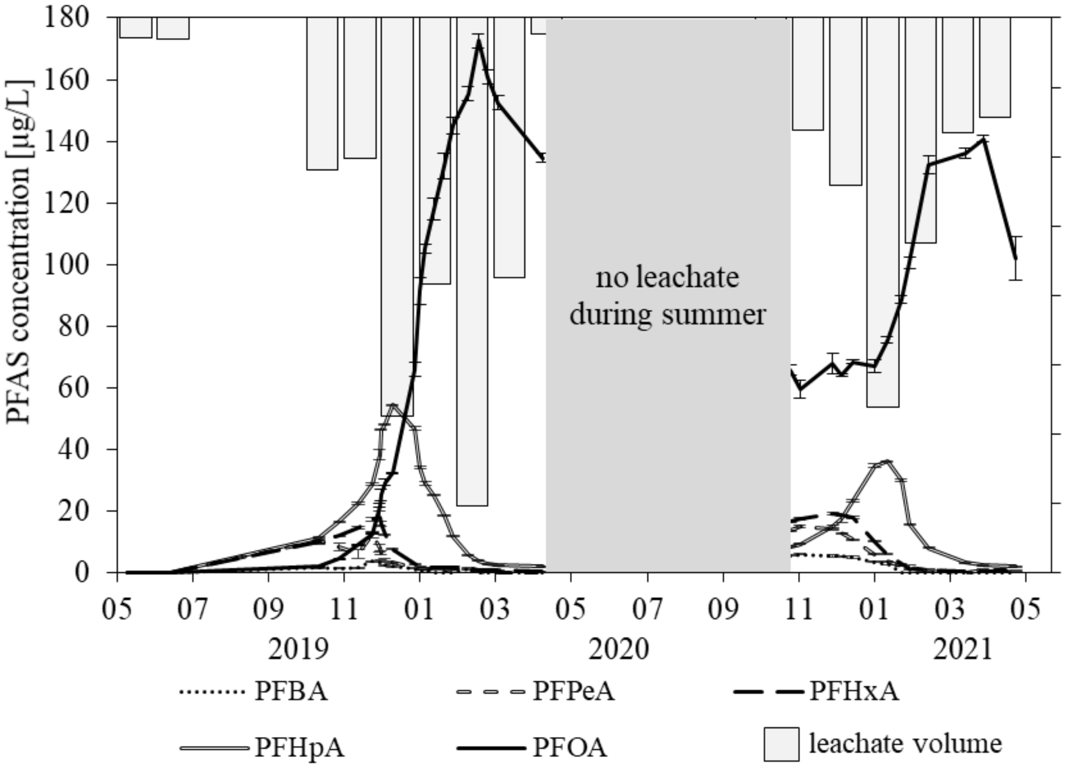

Figure S3: PFAS concentrations in the lysimeter leachate over time and monthly leachate volumes of the 8:2 diPAP variant; dTOP assay; mean value from 6 analytical replicates and standard deviation for PFAS concentrations.

$$DT_{50}[d] = \frac{\ln(2) \cdot t}{\ln\left(\frac{m_0}{m_t}\right)}$$

Equation S1: Calculation of the dissipation time of 50% (DT_{50}) assuming a first order kinetic model using the total experimental time (t), the total diPAP mass in the soil at the beginning of the study (m_0) and at the end of the study (m_t).

$$RR_{PFCA}\ [\%] = \frac{\sum n\ (PFCAs_{end})}{n\ (diPAPs_{start})\ \cdot\ 2} \cdot 100\%$$

Equation S2: Calculation of the recovery rate of PFCAs (RR_{PFCA}) from the degradation of diPAPs using the total molar substance amount (n) of diPAPs at the beginning of the study ($diPAPs_{start}$) and of PFCAs at the end of the study ($PFCAs_{end}$).

$$RR_{diPAP}\ [\%] = \frac{n\ (diPAPs_{end})}{n\ (diPAPs_{start})} \cdot 100\%$$

Equation S3: Calculation of the recovery rate of diPAPs (RR_{diPAP}) using the total molar substance amount (n) of diPAPs at the beginning of the study ($diPAPs_{start}$) and of PFCAs at the end of the study ($diPAPs_{end}$).
